# Reduced expression of N-Myc downstream-regulated gene 2 in human thyroid cancer

**DOI:** 10.1186/1471-2407-8-303

**Published:** 2008-10-22

**Authors:** Huadong Zhao, Jian Zhang, Jianguo Lu, Xianli He, Changsheng Chen, Xiaojun Li, Li Gong, Guoqiang Bao, Qiang Fu, Suning Chen, Wei Lin, Hai Shi, Jianjun Ma, Xinping Liu, Qingjiu Ma, Libo Yao

**Affiliations:** 1 Department of General Surgery, Tangdu Hospital, The Fourth Military Medical University. Xi'an, 710038, PR China; 2 Department of Biochemistry and Molecular Biology, The State Key Laboratory of Cancer Biology, The Fourth Military Medical University, Xi'an, 710032, PR China; 3 Department of Health Statistics, The Fourth Military Medical University, Xi'an, 710032, PR China; 4 Department of General Surgery, Xijing Hospital, The Fourth Military Medical University, Xi'an, 710032, PR China; 5 Department of Pathology, Tangdu Hospital, The Fourth Military Medical University, Xi'an, 710038, PR China; 6 Traditional Chinese Medical College, Xian'yang, 712000, PR China; 7 Department of Pharmacy, Xijing Hospital, The Fourth Military Medical University, Xi'an, 710032, PR China; 8 Department of neurosurgery, Xijing Hospital, The Fourth Military Medical University, Xi'an, 710032, PR China; 9 Department of Urology, Tangdu hospital, The Fourth Military Medical University, Xi'an, 710038, PR China

## Abstract

**Background:**

*NDRG*2 (N-Myc downstream-regulated gene 2) was initially cloned in our laboratory. Previous results have shown that *NDRG*2 expressed differentially in normal and cancer tissues. Specifically, *NDRG*2 mRNA was down-regulated or undetectable in several human cancers, and over-expression of *NDRG*2 inhibited the proliferation of cancer cells. *NDRG*2 also exerts important functions in cell differentiation and tumor suppression. However, it remains unclear whether *NDRG*2 participates in carcinogenesis of the thyroid.

**Methods:**

In this study, we investigated the expression profile of human *NDRG*2 in thyroid adenomas and carcinomas, by examining tissues from individuals with thyroid adenomas (n = 40) and carcinomas (n = 35), along with corresponding normal tissues. Immunohistochemistry, quantitative RT-PCR and western blot methods were utilized to determine both the protein and mRNA expression status of Ndrg2 and c-Myc.

**Results:**

The immunostaining analysis revealed a decrease of Ndrg2 expression in thyroid carcinomas. When comparing adenomas or carcinomas with adjacent normal tissue from the same individual, the mRNA expression level of *NDRG*2 was significantly decreased in thyroid carcinoma tissues, while there was little difference in adenoma tissues. This differential expression was confirmed at the protein level by western blotting. However, there were no significant correlations of *NDRG*2 expression with gender, age, different histotypes of thyroid cancers or distant metastases.

**Conclusion:**

Our data indicates that *NDRG*2 may participate in thyroid carcinogenesis. This finding provides novel insight into the important role of *NDRG2 *in the development of thyroid carcinomas. Future studies are needed to address whether the down-regulation of *NDRG*2 is a cause or a consequence of the progression from a normal thyroid to a carcinoma.

## Background

The well-known oncogene, *MYC*, was first identified as the cellular homolog of the viral oncogene myc [1]. Subsequent research showed that human cancers frequently display amplification of c-Myc, indicating the importance of this gene in cancer [2-4]. Expression of the c-Myc protein or the c-*MYC *gene is increased in a variety of human cancers, including over 80% of mammary cancers, 70% of colon cancers and 50% of hepatocellular carcinomas [5,6]. As an important oncogene and transcription factor, Myc was recognized as a dominant factor in cell cycle progression, cell differentiation, apoptosis and genomic instability. Because Myc promotes cell proliferation and inhibits cell differentiation [7,8], most of the target genes that are transcriptionally repressed by Myc have the opposite biological role. For example, they may inhibit cell proliferation, especially in cancer cells, or initiate cell differentiation.

Up-regulation of mouse *ndrg*1 was initially discovered in N-*myc *knockout mice [9]. Accordingly, it was named the 'N-*myc *downstream-regulated gene.' The mouse *ndrg *family now includes three members, *ndrg*1, *ndrg*2 and *ndrg*3. Subsequently, the human *NDRG *family members, *NDRG1*, *NDRG*2, *NDRG*3 and *NDRG*4, were cloned [10-13]. The amino acid sequence homology among human *NDRG *family members is 57–65%, indicating the conserved function of this family.

We were the first to identify human *NDRG*2 (AF 159092) and demonstrated that *NDRG*2 was a candidate tumor suppressor gene [10]. We also found that expression of *NDRG*2 in human glioblastoma tissues was significantly lower than in normal tissue and demonstrated that Myc repressed human *NDRG*2 through a Miz-1-dependent interaction with the core promoter of *NDRG*2 [14].

As a gene that is regulated downstream of Myc, *NDRG*2 expression has been shown to be reduced in many types of carcinomas. Our previous data and other reports showed that *NDRG*2 expression was decreased in breast cancer, lung cancer, hepatocellular carcinoma, colon cancer and gliomas [10,15-17]. Furthermore, *NDRG*2 was shown to be up-regulated in Alzheimer's brains [18] and could induce the differentiation of dendritic cells [19]. Our previous results also demonstrated the increased *NDRG*2 expression following the differentiation and maturation of U937 and HL60 leukemia cells. These findings implicate the important role of *NDRG*2 in cell growth and differentiation [14].

In the pathogenesis of thyroid cancer, evidence indicates that there are many genetic alterations and unique chromosomal rearrangements that occur, including the involvement of the RET-Ras-BRAF signaling cascade [20,21]. As human *NDRG*2 is important in cell proliferation and differentiation, we investigated whether *NDRG*2 participated in the carcinogenesis of thyroid cancer. Using immunochemistry, we first analyzed a tissue microarray of thyroid adenomas and carcinomas. The results showed that *NDRG*2 expression was significantly decreased in carcinomas, but was only slightly reduced in adenomas. We also detected obvious amplification of Myc in the thyroid cancer tissues, compared to normal tissues. This result was confirmed through a subsequent examination of clinical samples from patients with thyroid adenomas and carcinomas. Using real-time PCR and western blot analysis, we assessed the mRNA and protein expression levels of *NDRG*2 in thyroid carcinomas and adenomas.

## Methods

### 1. Tissue microarray

Two tissue microarrays, purchased from ChaoYing Biotechnology Co. Ltd. (CC15-11-001 and CC15-21-001), were used in the immunochemical analysis. Each microarray contains 159 thyroid tissue samples, including 81 thyroid carcinomas, 38 thyroid adenomas, and 40 normal thyroid tissues. Each thyroid tissue sample comes with detailed information regarding the patient, including gender, age and tumor histotype. Table 1 summarizes the data regarding the samples.

**Table 1:** List of various histotypes of thyroid tumor on the tissue arrays

	Papillary carcinomas	Follicular carcinomas	Medullary carcinomas	Undifferentiated carcinomas	Follicular Adenomas	Normal
	**n = 51**	**n = 15**	**n = 8**	**n = 7**	**n = 38**	**n = 40**
Women	41	9	6	3	33	25
Men	10	6	2	4	5	15
Age (Mean ± SD)	39.63 ± 16.07	39.37 ± 16.34	43.38 ± 16.34	43.63 ± 10.03	39.87 ± 11.46	40.78 ± 10.15

### 2. Clinical collection of thyroid samples

A total of 35 thyroid carcinomas samples were collected. This group was comprised of 20 papillary thyroid carcinomas (PTCs), eight follicular thyroid carcinomas (FTCs), five medullary thyroid carcinomas (MTCs), and two undifferentiated thyroid carcinomas (UTCs). Additionally, 40 thyroid adenoma samples were obtained. Human thyroid tumor tissues and their normal counterparts were accumulated between 2006 and 2007 from patients with thyroid tumors. They were gathered at the time of surgery from the Department of General Surgery, Tangdu Hospital and Xijing Hospital, FMMU (Xi'an, China).

The collection and use of human thyroid tissues were approved by the Fourth Military Medical University medical ethics committee. All patients provided written informed consent.

After surgical removal, all thyroid tumor tissues were immediately frozen in liquid nitrogen and stored at -70°C until processing. Each tumor was scored for clinical staging based on the tumor-node-metastasis (TNM) classification, which was introduced in 1987 by the International Union Against Cancer. All of the samples had been reviewed and given a final diagnosis by three different clinical pathologists. The distribution of gender, age, histological diagnosis and TNM stages for the 35 thyroid carcinomas is shown in Table 2.

**Table 2:** List of patients with thyroid carcinomas

ID	Age (yrs)	Sex	Histological diagnosis	pTxNxMx
1	39	male	PTC	pT1N0M0
2	45	male	PTC	pT2N1M0
3	56	male	PTC	pT1N0M0
4	25	female	PTC	pT1N0M0
5	27	female	PTC	pT3N0M0
6	28	female	PTC	pT1N0M0
7	30	female	PTC	pT1N0M0
8	31	female	PTC	pT2N0M0
9	32	female	PTC	pT1N0M0
10	36	female	PTC	pT2N0M0
11	36	female	PTC	pT1N0M0
12	38	female	PTC	pT1N0M0
13	39	female	PTC	pT2N0M0
14	40	female	PTC	pT4aN1M0
15	48	female	PTC	pT3N0M0
16	50	female	PTC	pT4aN1M1
17	52	female	PTC	pT1N0M0
18	55	female	PTC	pT1N0M0
19	59	female	PTC	pT1N0M0
20	67	female	PTC	pT1N0M0
21	67	male	FTC	pT1N0M0
22	27	female	FTC	pT2N0M0
23	38	female	FTC	pT1N0M0
24	40	female	FTC	pT1N0M0
25	45	female	FTC	pT1N0MX
26	56	female	FTC	pT4aN1M1
27	76	female	FTC	pT3N0M0
28	65	female	FTC	pT1N0M0
29	49	male	UTC	pT4aN1M0
30	59	male	UTC	pT4aN0M0
31	57	male	MTC	pT1N0M0
32	65	male	MTC	pT2N0M0
33	38	female	MTC	pT1N0M0
34	49	female	MTC	pT2N1M0
35	74	female	MTC	pT2N0M0

^a ^pT, pathologic tumor stage; N, nodal involvement; M, distant metastasis at the time of surgery.

PTC, papillary thyroid carcinoma; FTC, follicular thyroid carcinoma; UTC, undifferentiated thyroid carcinoma; MTC, medullary thyroid carcinoma.

### 3. Immunohistochemistry

A rabbit anti-human Ndrg2 polyclonal antibody was prepared in our laboratory. A PV-6000 immunohistochemistry kit was purchased from Zhongshan Company (Beijing, China). Paraffin wax and beeswax were purchased from Leica Histowax (Germany) and Hualing Company (Shanghai, China), respectively.

Tumor microarray paraffin blocks were cut into 4 μm sections. The sections were deparaffinized, heated at 70°C for 2 h, washed twice for 10 min in xylene, hydrated, and then washed twice for 3 min with phosphate-buffered saline (PBS). The tissue microarrays were incubated with 3% hydrogen peroxide for 10 min at room temperature and then subjected to three 3 min washes in PBS. Antigens were retrieved in 0.01 mol/L citrate buffer (pH 6.0) by incubating at 92–98°C for 25 min. Sections were allowed to cool naturally to room temperature and were washed twice for 3 min with PBS. The slides were then held in blocking solution for 30 min at 37°C. Sections were incubated with anti-Ndrg2 and anti-c-Myc polyclonal antibodies (1:50) for 4 h at 37°C and then washed with PBS three times for 3 min. Sections were incubated with HRP-conjugated goat anti-rabbit IgG secondary antibody for 10 min at 37°C. Immunolabeling was visualized by reaction with 3, 3'-diaminobenzidine (DAB). They were then removed from the DAB solution upon confirmation of color development and rinsed in PBS. Finally, the sections were counterstained with hematoxylin, dehydrated using graded dilutions of ethanol and coverslipped. A slide incubated with anti-actin primary antibodies served as the positive control, and a slide maintained in 0.01 mol/L PBS (pH 7.4), instead of the primary antibody solution, served as a negative control.

### 4. Immunohistochemistry scoring criteria

To examine the immunohistochemistry, we used the scoring method of Sinicrope et al. [22]. Both the intensity and the extent of immunological staining were analyzed semi-quantitatively. Array fields were scored with respect to the quantity of positive cells. Sections with no labeling or with fewer than 5% labeled cells were scored as 0. Sections were scored as a 1 with labeling of 5–25% of cells, as a 2 with 25–50% of cells and as a 3 with 50–75% of cells. Finally, labeling of ≥75% of the cells was scored as a 4. The staining intensity was scored similarly, with 0 used for negative staining, 1 for weakly positive, 2 for moderately positive and 3 for strongly positive. The scores for the percentage of positive tumor cells and for the staining intensity were multiplied to generate an immunoreactive score for each specimen. The product of the quantity and intensity scores were calculated such that a final score of 0 indicated no expression, 0–4 indicated weak expression, 4–8 indicated moderate expression and 8–12 indicated strong expression. Each sample was examined separately and scored by two pathologists. Cases with discrepancies in the scores were discussed to reach a consensus.

### 5. RNA isolation and quantitative RT-PCR analysis

The tissue had been frozen in liquid nitrogen and stored at -80°C. Total RNA was extracted and purified from each sample, using the TRIzol reagent (Invitrogen). After quantification, total RNA (2 μg) was reverse-transcribed with reverse transcriptase (Promega, WI), according to the manufacturer's instructions. All PCR experiments were performed with Taq polymerase (Promega, WI). Quantitative RT-PCR was performed on a Prism 7500 real-time PCR system (Applied Biosystems) in Universal Mastermix (Applied Biosystems) to amplify *NDRG*2 and c-*MYC*. Primers were designed using the Primer Express Software (ABI, Foster City, CA). SYBR green (TAKARA) was used in each reaction for detection. The *NDRG*2 primers were 5'-GCCCAGCGATCCTTACCTACC-3' and 5'-GGCTGCCCAATCCATCCAACC-3'. The c-*MYC *primers were 5'-GGAGGAACAAGAAGATGAGGAAG-3' and 5'-AGGACCAGTGGGCTGTGAGG-3'. The *GAPDH *primers were 5'-GCCTCAAGATCATCAGCAAT-3' and 5'-AGGTCCACCACTGACACGTT-3'. *NDRG2*, c-*MYC *and *GAPDH *mRNAs were quantified separately in triplicate.

Each sample was used in a single reaction, which was incubated at 95°C for 10 s, followed by 40 cycles of 95°C for 5 s and 60°C for 34 s. Fluorescent data were converted into cycle threshold (CT) measurements, using the ABI Prism 7500 SDS system software.

### 6. Cell lysis and western blot analysis

Cells from each tissue sample were washed twice with ice-cold phosphate-buffered saline. Cells were collected and lysed with lysis buffer (50 mM Tris, pH 7.5, 150 mM NaCl, 1 mM MgCl_2_, 0.5% NP-40, 0.1 mM PMSF and protease inhibitor cocktail). Whole protein lysate (50 μg, measured using the BCA protein assay, Pierce, Rockford, IL) was loaded onto 12% SDS polyacrylamide gels for electrophoresis and transferred to Hybond ECL nitrocellulose membranes (Amersham Biosciences, Piscataway, NJ). The membranes were blocked with 5% fat free milk in Tris-buffered saline containing 0.05% Tween-20 and incubated with each primary antibody (Ab) overnight at 4°C. The following primary antibodies were used: anti-Ndrg2 goat polyclonal (Santa Cruz Biotechnology, Santa Cruz, CA), anti-c-Myc rabbit polyclonal (sc-764; Santa Cruz Biotechnology) and anti-β-actin rabbit polyclonal (Boster, Wu Han, China). Finally, to detect the primary antibody, blots were incubated with horseradish peroxidase-conjugated secondary antibodies (1:4000 dilution; Santa Cruz Biotechnology) for at least 1 h at room temperature. The ECL detection solutions (Pierce) were then applied. Scanned images were quantified using the Kodak Digital Science 1D software (Kodak, New Haven, CT).

### 7. Statistical analysis

For real-time PCR and immunostaining assays, significant differences across groups were analyzed using the Kruskal-Wallis tests and the Wilcoxon rank sum tests with the Bonferroni's correction for pairwise comparisons. Comparisons between cancer (adenoma) tissues and tissue adjacent to the cancer (adenoma) were performed using the Wilcoxon signed-rank test. The correlation analysis between RNA and protein was calculated using the Spearman correlation coefficient, and this analysis was used as well for Myc and *NDRG*2. All tests were two-tailed, and statistical significance was set at *p *< 0.05.

## Results

### 1. Differential expression of Ndrg2 in thyroid carcinomas and normal tissues

**Figure 1:**
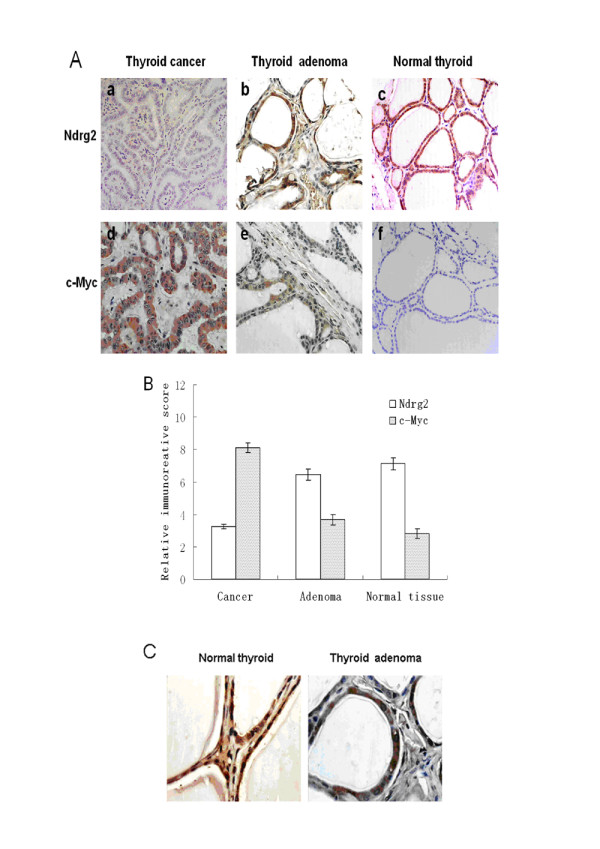
**Differential expression of Ndrg2 and c-Myc in thyroid carcinoma, adenoma and normal tissue**. (A) Immunohistochemical analysis of Ndrg2 and c-Myc in thyroid cancer, adenoma and normal tissues. (B) Ndrg2 expression was higher in normal tissue than the carcinomas (p < 0.05). The amplification of c-Myc was more abundant in cancers than in normal thyroid tissue. (C) Analysis of Ndrg2 localization in thyroid cells. The brown immunostaining indicates the cytoplasmic localization of Ndrg2.

Because Ndrg2 expression is decreased in many types of cancer tissues [10,15], a preliminary analysis on Ndrg2 expression in thyroid tumors was performed using a tissue microarray. With immunostaining analysis, a different expression pattern of Ndrg2 was detected in thyroid carcinomas and normal tissues (Fig. 1A). By pairwise comparison with Bonferroni's correction, we found a statistically significant increase of Ndrg2 expression in normal thyroid compared to carcinomas (Fig. 1B). We also noted that Ndrg2 expression decreased slightly in thyroid adenomas, though the difference was not statistically significant (*p *> 0.1).

Our previous results showed that Ndrg2 was primarily localized in the cytoplasm of mouse tissues [23]. In human thyroid tissue, we also detected predominantly cytoplasmic localization of Ndrg2 in both normal and adenoma tissues (Fig. 1C). Substitution of the primary antibody with a control serum abolished immunostaining in the tissue microarray (data not shown).

We confirmed that Myc could transcriptionally repress the expression of human *NDRG*2 during the processes of cell proliferation and differentiation [14]. We also examined the expression level of c-Myc in the thyroid tissue microarray. Interestingly, the results were similar to those observed in gliomas [10], with amplification of Myc in thyroid cancers, compared to normal tissues (Fig. 1A, C).

### 2. Decreased Ndrg2 protein level in clinical samples of thyroid tissue

The expression profiles of Ndrg2 in the microarray of thyroid tissues suggested an important role for Ndrg2 in thyroid carcinogenesis. We thus wanted to analyze the expression level of Ndrg2 protein in adjacent normal and neoplastic thyroid tissues from human patients to confirm the results. The thyroid cancer and corresponding normal samples on the microarray were not paired well. Therefore, we had extra incentive to collect paired thyroid carcinomas (n = 35), adenomas (n = 40) and their normal counterparts. We then examined the expression profiles of Ndrg2 and c-Myc by immunohistochemistry and western blotting.

**Figure 2:**
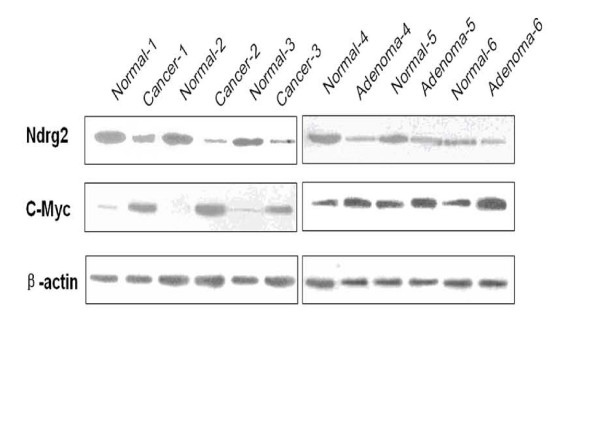
**Expression of Ndrg2 and c-Myc protein by Western blot analysis in cancer, adenoma and normal thyroid tissues**. Three paired thyroid cancers and the normal counterpart, along with three thyroid adenomas and the normal counterpart were analyzed by Western blotting. Anti-Ndrg2 and anti-c-Myc antibodies were used, while an anti-β-actin antibody was the control.

The immunostaining results demonstrated the same pattern of expression as the microarray (data not shown). Furthermore, the protein levels of Ndrg2 and c-Myc were analyzed using western blotting. Expression of Ndrg2 was reduced in thyroid cancers, compared to normal tissues (Fig. 2), consistent with the microarray analysis. We also observed reduced expression of Ndrg2 protein in a few samples of thyroid adenomas. Compared with the corresponding normal tissues, positive amplification of c-Myc was detected in the thyroid cancers, while little c-Myc was seen in the thyroid adenomas. These results suggest that decreased expression of Ndrg2 correlates with thyroid cancer.

### 3. Reduced *NDRG*2 mRNA level in thyroid cancer

**Figure 3:**
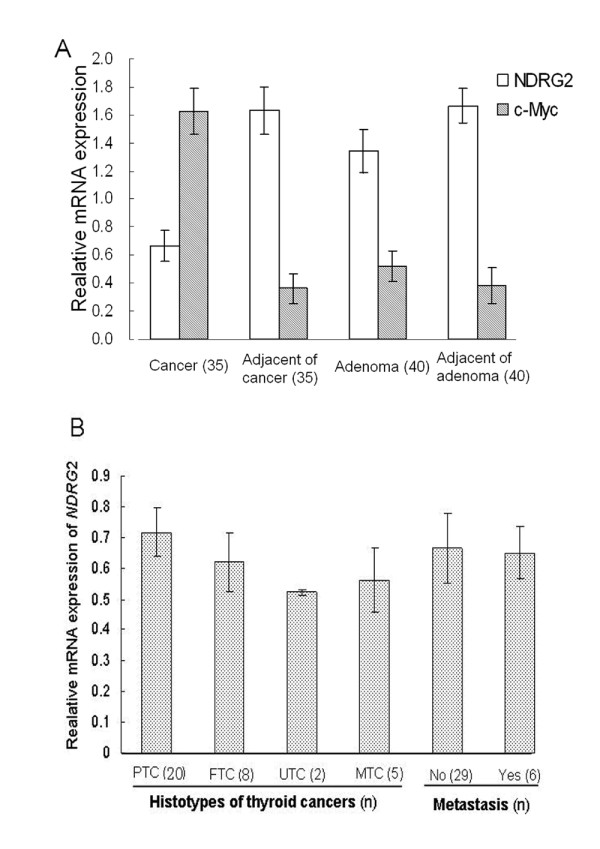
**Reduced *NDRG*2 and increased c-*MYC *mRNA level in thyroid cancers**. (A) Real-time PCR was used to detect differences in *NDRG*2 and c-*MYC *RNA levels in thyroid tissues. The mean values of *NDRG*2 and c-*MYC *mRNA levels were calculated after normalization to the expression of *GAPDH*. The P values comparing the expression of *NDRG*2 and c-*MYC *between the thyroid cancer and the adjacent normal tissues were below 0.05. (B) Analysis of *NDRG*2 expression using the different histotypes and distant metastasis of thyroid cancer. There are four histotypes of thyroid cancers, including papillary carcinomas (PTC), follicular carcinomas (FTC), medullary carcinomas (MTC) and undifferentiated carcinomas (UTC). N in the bracket indicates the numbers of each histotype. There were six patients with distal metastases and 29 with no metastasis. *p *> 0.05.

Based on the detection of Ndrg2 protein expression in thyroid cancer tissues, real-time RT-PCR was used to investigate *NDRG*2 mRNA levels in thyroid adenomas and carcinomas. The data showed that expression of *NDRG*2 mRNA in thyroid cancer tissues was lower than in the adjacent cancer group (Fig. 3A). Expression of *GAPDH *was used for normalization. However, the expression of c-Myc was extremely high in cancer tissues. Upon examining these levels of expression, we noted a tendency towards decreased *NDRG*2 expression with increasing c-Myc expression in thyroid cancer (Fig. 3A). There was a negative correlation between Myc and *NDRG*2 expression levels in the thyroid carcinomas (r = -0.336, p = 0.048).

Further analysis of *NDRG2 *and c-Myc expression in adenomas and the adjacent tissue showed no significant differences. Moreover, we analyzed the association of *NDRG*2 expression with the patient's gender and age. Dividing all of the thyroid tissue samples into male and female groups revealed mostly balanced levels of *NDRG2 *expression for both groups, but with slightly lower *NDRG*2 expression in the females (Table 3). Furthermore, *NDRG*2 expression was slightly decreased in the group of patients who were over 45 years, compared to the group younger than 45 (Table 3). However, this difference was not statistically significant. Thus, expression of *NDRG*2 did not correlate with gender or age (Fig. 3B). Finally, potential correlations between *NDRG*2 mRNA expression, the different histotypes of thyroid cancers and distant metastases were investigated. We found no significant differences between the groups.

**Table 3:** Mean values of normalised levels of *NDRG2 *mRNA in thyroid cancer, adenoma and normal tissues with different ages and gender groups

	**Cancer**		**Adenoma**		**Adjacent of cancer**		**Adjacent of adenoma**	
				
	**n**	**$\bar{x}$ ± *sd***	**n**	**$\bar{x}$ ± *sd***	**n**	**$\bar{x}$ ± *sd***	**n**	**$\bar{x}$ ± *sd***
**Age (years)**								
**< 45**	16	0.68 ± 0.106	26	1.33 ± 0.71	16	1.609 ± 0.194^a^	26	1.658 ± 126^c^
**≥45**	19	0.650 ± 0.099	14	1.353 ± 0.128	19	1.650 ± 0.153^a^	14	1.676 ± 126^c^

**Gender**								
**Male**	8	0.718 ± 0.099	4	1.367 ± 0.179	8	1.609 ± 0.088^b^	4	1.635 ± 0.107^d^
**Female**	27	0.648 ± 0.0106	36	1.337 ± 0.155	27	1.638 ± 0.190^b^	36	1.668 ± 0.128^d^

^a ^corrected *p *< 0.05 compared with cancer tissue from the same age group.

^b ^corrected *p *< 0.05 compared with cancer tissue from the same gender group.

^c ^corrected *p *> 0.05 compared with adenoma tissue from the same age group.

^d ^corrected *p *> 0.05 compared with adenoma tissue from the same gender group.

## Discussion

The annual occurrence of thyroid cancer varies considerably in different regions, ranging from 1.2–2.6 per 100,000 individuals in males and from 2.0–3.8 per 100,000 in females. Several reports have shown that the incidence of thyroid cancer has increased in recent years [20,24]. With better diagnostic methods, there is also more detection of thyroid cancers at earlier stages. However, some thyroid cancer still develops into advanced stages with spreading and metastasis. It remains unclear how normal thyroid tissue develops into cancerous tissue.

In the present study, we demonstrated that the mRNA and protein expression levels of *NDRG*2 were decreased in thyroid cancers, compared to normal tissues. We first observed the different expression profiles of Ndrg2 in a thyroid tissue array using immunohistochemistry, and we found low expression levels of Ndrg2 in the carcinomas, compared with normal tissue. In accord with our previous finding that Myc can transcriptionally repress human *NDRG*2, we also detected amplification of c-Myc in thyroid cancers, consistent with findings in other cancers [5,6].

To examine Ndrg2 at the levels of mRNA and protein, we analyzed a collection of clinical samples, including 35 thyroid cancers and 40 adenomas. Our results showed significantly reduced expression of Ndrg2 in thyroid cancers, indicating the potential involvement of *NDRG*2 in the processes of thyroid carcinoma formation. Real-time PCR results demonstrated no correlation between *NDRG*2 expression and the patient's age or gender. There was also no clear difference among the various types of thyroid cancer. A slight decrease in *NDRG*2 was detected in the thyroid adenoma tissues.

Our data indicated there was no correlation between Ndrg2 and the metastasis of thyroid cancers. This result was similar to the finding of Jung SH in breast cancer [25]. This group detected an inverse correlation between Ndrg2 expression and breast tumor size, but found no relationship with auxiliary lymph node metastasis. As we did not define the role of Ndrg2 in the metastasis of thyroid cancer, the mechanism of the involvement of Ndrg2 in cancer needs to be more thoroughly examined in future studies.

We initially identified human *NDRG*2 as a candidate tumor suppressor gene [10]. We found that the expression of *NDRG*2 was significantly reduced in human glioblastoma tissues. Although the slight decrease of *NDRG*2 expression in thyroid adenomas was not significant, it suggests that *NDRG*2 might be involved in mediating the progression from thyroid adenoma to carcinoma. Furthermore, *NDRG*2 expression increased following the differentiation of colon and dendritic cells [19]. Over-expression of *NDRG*2 caused an inhibition of cell proliferation and increased apoptosis in gastric cancer, through Fas-mediated cell death [26]. *NDRG*2 may be involved in tumor progression and overall survival of gastric cancer patients.

Whether the down-regulation of *NDRG2 *in thyroid carcinoma is a cause or a consequence remains presently unclear. Further studies are needed to investigate how *NDRG2 *is involved in the progression from normal thyroid tissue to thyroid cancer.

## Conclusion

In conclusion, the protein and mRNA expression levels of *NDRG*2 were significantly decreased in thyroid cancers with c-Myc amplification. However, there were no significant correlations of *NDRG*2 expression with gender, age, the different histotypes of thyroid cancers, or distant metastases. Our data provide novel insight into the important role of *NDRG2 *in the development of thyroid cancers. Further studies are needed to address whether the down-regulation of *NDRG*2 is a cause or consequence of the progression from the normal thyroid tissue to a carcinoma.

## Competing interests

The authors declare that they have no competing interests.

## Authors' contributions

LY, QM and XL conceived the idea of the study. HZ, JZ and QF designed the primers, extracted the RNA and carried out the quantitative RT-PCR. JL, XH and XL collected the thyroid samples. LG, JM and GB were responsible for the immunochemical analysis of the thyroid tumor samples and the tissue array. SC, WL and HS carried out the cell lysis and western blot analysis. CC was responsible for statistical analysis. HZ drafted the manuscript. JZ helped write the manuscript. All authors contributed to interpretation and discussion of the results. They also read and approved the final version.

## Pre-publication history

The pre-publication history for this paper can be accessed here:


